# Global bioethics: it’s past and future

**DOI:** 10.1080/11287462.2021.2011009

**Published:** 2022-02-08

**Authors:** Cheryl Macpherson

**Affiliations:** a Department of Clinical Skills, Bioethics Division, St George's University, St George's, Grenada; b Windward Islands Research and Education Foundation (WINDREF), Grenada

A google scholar search for “global bioethics” returns citations situating ethical analyses within the evolving social and physical features of the global environment. Such work is consistent with Van Rensselaer Potter’s “Global Bioethics” (1988) and its application of bioethics to global issues of health and human survival such as nuclear war and what he called “global warming”. Ruth Macklin, in this special issue (SI), offers the Covid-19 pandemic as such an issue and delineates global bioethics from “international bioethics” which address country-specific issues isolated from wider global influences. A search for “global bioethics” in non-academic search engines returns items about international bioethics, and not global bioethics. This editorial concludes this special issue (SI) by underscoring links between its contents and the history of global bioethics, offering a view to the future of global bioethics as a field and future aims and scope of this journal. It turns out that the field contributed to the establishment of this journal although it’s contents shifted over time into Macklin’s international bioethics.

This SI examines what global bioethics might bring to the often distressing global environments of the 2020’s and associated animosities, misinformation, and information overload. It’s authors show how globalization influences health and increases dependence of individuals and populations on global systems for essential resources: food, water, shelter, air, and more. Globalization seems driven primarily by industrial and corporate entities that have wealth and power. In maximizing growth and profit for their shareholders, they may choose to embrace health-promoting or health-harming strategies and policies. Authors herein suggest that examining competing interests like these in a global (as well as international and local) context may increase transparency and better inform decision-making and policies to protect human health and survival long into the future.

Several things surprised me in the process of reading the contents of this SI. One was Rosemary Tong’s explicit wish that she had paid more attention to Potter’s views early in her career. Another was something she and others herein state that I had not known - that Potter’s views contributed to establishing the International Association for Bioethics (IAB), International Association for Feminist Approaches to Bioethics (FAB), and International Bioethics Committee (IBC). Like Macklin, Tong turns to pandemic planning and cooperation while offering some history. Tong hopes “for a future, care-based feminist global bioethics … [because] unless we human beings learn how to care for each other … we cannot hope to respect each other’s rights” or protect and share resources essential for human health and survival. Macklin pragmatically concludes that diplomacy is “a necessary ingredient” in engaging with “ethical aspects of relations between and among nations or regions”. Effective diplomacy must surely require both care and respect, making their views synergistic.

In his IAB presidential address on global bioethics, Alistair Campbell (1999) called for greater attention to social and political influences in and on health and healthcare, greater diversity of methods used, and greater critique of Western norms and approaches which tacitly support “the idea of constant economic progress as an end for humanity” (pp. 3–4). Prescient to the globalized and counterfactual 2020’s he noted that “not all ethics talk is of equal validity” (pp. 2–3). Historic accounts of bioethics by Henk Ten Have (2012) and others describe the rise of influential bioethics centers in the United States and other Western democracies. Their work reflected emerging issues in their countries, namely technology and autonomy in patient care, and misconduct and conflicts of interest in research. This prioritized individual choice and sidelined attention to social and physical (including geographic) influences on the choices available to individuals.

The origins of global bioethics is recounted by authors including Peter Whitehouse (2003) who wrote that “the future of bioethics lies to a considerable degree in its past”. Attempting to advance Potter’s ideas, Whitehouse writes that our words, ideas, and actions alter the noosphere, a concept discussed by Pierre Teilhard de Chardin (1881–1955) and prior philosophers involving the influence of human thought on the biosphere^1^. In his discussion of globalization, Gustavo Ortiz Millam (SI) gives life to this concept in saying that “what happens in one part of the world shapes events occurring at the same time somewhere else” and accentuates interdependency and interconnections of individuals and their choices.

The noosphere concept highlights serendipitous (or perhaps coincidental) aspects of my career that connect with the history and future of global bioethics. Whitehouse (2001) distinguished global bioethics from other forms of bioethics by its concern with planetary aspects of health and the future, and suggested that the American Society for Bioethics and Humanities (ASBH) should adopt ecofriendly policies for its conferences to demonstrate this concern. The suggestion was reiterated by others some years later (personal communication), and again as a formal request to the ASBH president (2018–2019) by its Environmental Ethics interest group which I belonged to for several years. The cursory response received contributed to my later decision not to renew my ASBH membership. Led by Cristina Richie in 2020, the group submitted (again) a similar petition to the board with its annual report “Recommendations for a More Sustainable ASBH” that drew from ASBH strategic domains and strategic initiatives, receiving no response.

The importance of planetary systems for human health and long-term survival is evident in *the Lancet Planetary Health* journal and the “Pledge for planetary health to unite health professionals in the Anthropocene” co-authored by epidemiologist and public health leader Sir Andrew Haines (https://www.thelancet.com/article/S0140-6736(20)32039-0/fulltext). These strengthen the claim by Whitehouse that planetary systems are bioethics concerns. Potter (2001), when discussing the diversity of sources that informed his ideas, includes a book co-authored by Haines with physician-environmentalist Eric Chivian and others. A 2008 lecture by Haines first showed me that policies permitting climate change to continue are bioethics concerns and motivated my work on the topic, some of which was published in the journal *Public Health Ethics* (Macpherson & Akpinar-Elci, 2015). The editors of that journal are Angus Dawson (whose words are considered below) and Marcel Verweij.

Dawson (2010) reflects that commercialization influences health and that public health ethics may help turn bioethics toward individuals as parts of communities, populations, and wider environments. He writes that “Not least of the issues here is how an individual's health may be thought to be a point *within* a population's health, where your health is at least partly determined by the social and political environment within which you live as well as how healthy others are within the population you inhabit. Once again, many of the relevant factors lie beyond individual autonomous control, and push instead towards other values such as equity and solidarity.” He never cites Potter or global bioethics but the core of global bioethics supports his thesis:

“ … the choices that we make in relation to food should not be thought of using some naïve model of autonomous agency, but should take account of the influences of commercial, political and cultural decisions that go beyond the autonomous decisions of individuals. Food choices require us to think about how the features of a capitalist free market influence decisions about what we eat (e.g. advertising and fashion), about subsidies to farmers that result in surplus corn being converted into high-calorie additives in processed food, and the way that fewer people know much about how to grow or cook food, etc. To think of rising rates of obesity as though they were solely the result of weak-willed individuals or a life-style choice is again naïve. The best way for you to live your life, and to enjoy your autonomy, is to live well with others.”

Maintaining a global rather than international perspective, Sirkku Hellsten (2008) highlights differences involving geographies, economies, and countries that influence human health and survival. Henk Ten Have (2012) discusses Potter’s ideas as they evolved with time and changing social and physical concerns at global, and local, levels. Unrestrained global population growth troubled Potter, for example, and was a social concern in the 1960’s due to its depletion of natural resources essential for human health and survival (p. 63); by the 1980’s, Potter saw bioethics as attentive to “old problems” like abortion rather than new ones involving human survival, and as reliant on philosophical rather than approaches from arts and sciences (p. 75). Potter (2001) lived to see these ideas integrated into the UN Millennium Goals which he described as an ethical statement obligating all professionals, particularly its signatories, to advance bioethics and sustainability. That these ideas, as ten Have and Tong each note (SI), were later integrated into UNESCO’s *Universal Declaration on Bioethics and Human Rights* attest to their ongoing value globally and for bioethics.

In his final years, Potter (2001) hoped that global bioethics, or a new field, would seek wisdom with which “to use biological knowledge for the social good” (p. 20). He wrote that professionals, once perceived as wise trustees of public good, are increasingly seen as technical experts with little wisdom beyond their specialties. He defined civil society and social responsibility as essential to health and survival, ideas echoed by Bruce Jennings and others in a special issue of the *Hastings Center Report* (2021) addressing the future of democratic societies. Potter (2001) specified global population growth, economic and gender disparities, and environmental change as dilemmas for global bioethics. Twenty years later, these unresolved dilemmas significantly threaten health and survival.

Potter was indirectly involved in establishing the journal *Global Bioethics*. Brunetto Chiarelli, an Italian anthropologist named on the website as its founding editor, had admired Potter’s global bioethics work and, over time, became his friend and then partner in establishing the “global bioethics network”^2^ in which they were soon joined by Whitehouse and others from Mexico, Japan, and Spain (Erin Williams, 2001). The first issues of the journal (1988–1991) are available online but only in Italian (https://www.tandfonline.com/doi/abs/10.1080/11287462.1991.10800569). A more complete history of the journal and network, and subsequent work by network members, might find interesting influences of global bioethics in other disciplines, and point toward new understandings and resolutions for today’s dilemmas. It might better engage those in diverse fields in elucidating ways to protect health and survival from climate change. Globalization and global population growth increase global demand for resources, and industries release more emissions in meeting that demand, this perpetuates climate change with its damage to health and resources.

Initially committed to global bioethics, the focus and content of the journal *Global Bioethics* drifted at some point to international bioethics (reports of concerns in different countries). These exposed neglected perspectives and challenges (often from the Global South) that warrant attention in Western bioethics and care (Tong) from everyone. The story by Christine Overall (SI) about Indigenous women singing to heal themselves, as well as those who harmed them, is an example of care and Overall’s intersectional approach to global bioethics seems consistent with Potter while adding perspective to Tong. Global bioethics analyses of “off the beaten track” perspectives and challenges may nurture solidarity and the cooperation necessary to designing and implementing effective local and global responses to global problems.

Food, water, air, land offered by the Earth, atmosphere, and planetary systems are biologically and geographically limited. Human health and ethics evolved from what began as a small population on an Earth of vast resources to a population of billions whose essential needs exceed these resources. This is captured by the biological reality of carrying capacity and the conceptual and practical realities resulting from any tragedy of the commons (Garvey, 2008; Hardin, 1968). Historically, governments have responsibilities for the health and wellbeing of their citizens, and industries and their leaders have civic responsibilities to their governments and communities including consumers. Governments and industries have the greatest control over Earth’s environments and resources but continue to fail in stewarding these for citizens and consumers. Unless they adhere to agreements made during the 2021 COP-26 in Glasgow there is little hope for health and survival in the near or distant future. Global bioethics work might motivate helpful shifts in their economic priorities and practices. It might ask whether economics or politics justifiably take priority over health and human survival, and to why governments or industries would permit these to do so. The integration of Potter’s ideas into the UN Millennium Goals and more recent Sustainable Development Goals hints at their resurgence and may blossom into a healthy sustainable future for humanity (or not).

In 2022, expect the new co-editors Richie and Macpherson and the new Editorial Board of the journal *Global Bioethics* to re-shape the journal’s aims and scope in light of this SI. Meanwhile, we will continue to review and publish submissions while encouraging potential authors to situate their work in a global context. We particularly welcome submissions from the Global South and from Potter’s former colleagues, co-authors, critics, mentees, and members of the global bioethics network, and open an additional call for submissions on the commentaries in this SI.

## Notes

1 According to Ten Have (2012) Chardin’s work greatly influenced Potter as did, as an aside, Margaret Mead’s.

2 The ‘global bioethics network’ formed by Potter is not the ‘global health bioethics network’ based at Oxford University and listed on the journal’s website.
